# Mixed Olfactory Neuroblastoma and Adenocarcinoma with In Situ Neuroendocrine Hyperplasia

**DOI:** 10.1007/s12105-019-01062-w

**Published:** 2019-08-06

**Authors:** Jonathan E. Attwood, Deva Sanjeeva Jeyaretna, Fintan Sheerin, Ketan A. Shah

**Affiliations:** 1 Nuffield Department of Clinical Neurosciences, John Radcliffe Hospital, University of Oxford, Level 6 West Wing, Headley Way, Oxford, OX3 9DU UK; 2 Department of Neurosurgery, John Radcliffe Hospital, Oxford, UK; 3 Department of Neuroradiology, John Radcliffe Hospital, Oxford, UK; 4 Department of Cellular Pathology, John Radcliffe Hospital, Oxford, UK

**Keywords:** Esthesioneuroblastoma, Olfactory neuroblastoma, Neuroendocrine carcinoma, Adenocarcinoma, Nasal cavity

## Abstract

Olfactory neuroblastoma (ONB) is a rare malignant neoplasm arising from the superior aspect of the nasal vault. Cases are characterised by insidious clinical presentation and high rates of recurrence despite surgical resection and adjuvant radiotherapy. There are a small number of reports showing ONB with divergent epithelial or ganglionic differentiation, and ONB has also been found to coincide with adenocarcinoma. We present a case of mixed ONB with adenocarcinoma. The clinical presentation was unusual, with a tonic–clonic seizure preceded by chronic headache and anosmia. Imaging revealed a mass extending from the olfactory recess of the left nasal cavity through the cribriform plate to the anterior cranial fossa. The pathology demonstrated intraepithelial neuroendocrine cell hyperplasia in the left olfactory groove. This finding provides a unique insight into the cellular origin of this rare tumour, and appears to confirm the theory that ONB arises from neural stem cells in the olfactory neuroepithelium. Despite radical treatment, the patient suffered a distant recurrence within 1 year of treatment, which underlines the aggressive nature of this tumour.

## Introduction

Olfactory neuroblastoma (ONB), or esthesioneuroblastoma, is a rare malignant neoplasm arising from the superior aspect of the nasal vault [1]. ONB was first described by Berger et al. in 1924 [2], and is thought to originate from pluripotent neural stem cells which supply the olfactory neuroepithelium lining the underside of the cribriform plate of the ethmoid bone [1]. The tumour accounts for up to 3% of intranasal neoplasms [3] with peak incidence in the fifth or sixth decade and a slight male predominance (55%) [1].

Cases of ONB are characterised by insidious growth and slow onset of symptoms, leading to a delay in diagnosis of 6–12 months from the time of symptom onset [1]. The most common presenting symptoms are unilateral nasal obstruction and epistaxis, followed by headache, sinusitis, anosmia, and, more rarely, visual disturbance, pituitary dysfunction, or neurologic symptoms secondary to intra-orbital or intra-cranial invasion [1]. CT and MRI imaging of the head and neck are used to stage disease, after which intra-operative biopsy is required for a histopathological and immunohistochemical diagnosis.

The mainstay of treatment for ONB is surgical resection. Adjunctive radiotherapy is offered to most patients, while chemotherapy is thought to provide benefit only in the most advanced disease. Despite treatment, patients remain at relatively high risk of recurrence, with local, regional, and distant recurrence rates of 29%, 16%, and 17% respectively [4]. 5-year survival rates range from 75% in cases where tumour is confined to the nasal cavity (Kadish Stage A) to 41% when tumour invades beyond the sinonasal cavities (Kadish Stage C) [5].

Unlike peripheral neuroblastomas, ONB is known to occasionally show divergent epithelial or ganglionic differentiation. This can occur spontaneously [6–14] or in response to chemotherapy [15] and radiotherapy [16]. The diversity of this tumour is likely to be result of the high degree of pluripotency shown by the neural stem cells from which it is thought to arise. ONB has also been found to coincide with adenocarcinoma, first described by Miller et al. in 1984 [6], and in a further three cases reported by Seethala et al. in 2007 [13].

In this report, we present a case of mixed ONB with adenocarcinoma which demonstrates intraepithelial neuroendocrine cell hyperplasia in the left olfactory groove. This finding provides a unique insight into the cellular origin of this rare tumour, and appears to confirm the theory that ONB arises from neural stem cells in the olfactory neuroepithelium.

### Case Presentation

A 66-year-old right-handed female presented to the Emergency Department after her first generalised tonic–clonic seizure that had started while she was sitting watching television. Four months prior to this, the patient had experienced a single absence-like seizure episode described as a short period of staring without being able to speak. There was also a nine-month history of mild early morning headaches and intermittent anosmia. Her past medical history included choriocarcinoma in remission, coeliac disease, hiatus hernia, osteopaenia and allergic rhinitis. Examination of the head, neck and neurological system was normal. Full blood count, coagulation, liver function, urea and electrolytes were within normal ranges, except for a moderate hyponatraemia (122 mmol/L), low serum osmolality (263 mOsm/kg) and low urine osmolality (134 mOsm/kg), consistent with a syndrome of inappropriate antidiuretic hormone secretion (SIADH).

**Fig. 1:**
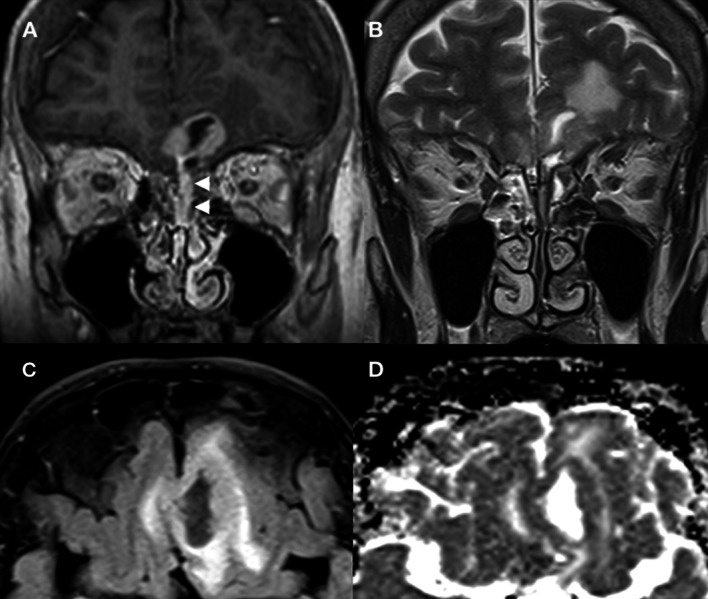
MRI of the anterior skull base. Coronal T1 post-contrast (**a**) and coronal T2 (**b**) demonstrate a mixed solid cystic mass extending from the olfactory recess of the left nasal cavity (white arrowheads) through the left cribriform plate and into the anterior cranial fossa. The mass is adhered to the pial surface of the frontal lobes of the brain bilaterally. There is adjacent vasogenic oedema within the brain parenchyma on the coronal T2 (**b**) and axial FLAIR (**c**) sequences. The solid portions demonstrate avid enhancement (**a**) and intermediate to restricted apparent diffusion (**d**) consistent with a cellular neoplasm

MRI scan of the head revealed a cystic mass extending from the left nasal cavity into the anterior cranial fossa, associated with vasogenic oedema at the base of both frontal lobes (Fig. 1). There was minor midline shift to the right and minimal effacement of the anterior horn of the left lateral ventricle, but no evidence of hydrocephalus or haemorrhage. The patient was given dexamethasone for reversal of cerebral oedema and levetiracetam for seizure prophylaxis. Computed tomography (CT) scan of the thorax, abdomen and pelvis showed no evidence of disseminated malignancy (modified Kadish stage C, Dulguerov stage T4N0M0, Biller stage T3N0M0). The case was discussed in the neuro-oncology multi-disciplinary team meeting, and the decision was taken to offer an endoscopic-guided anterior skull base biopsy.

**Fig. 2:**
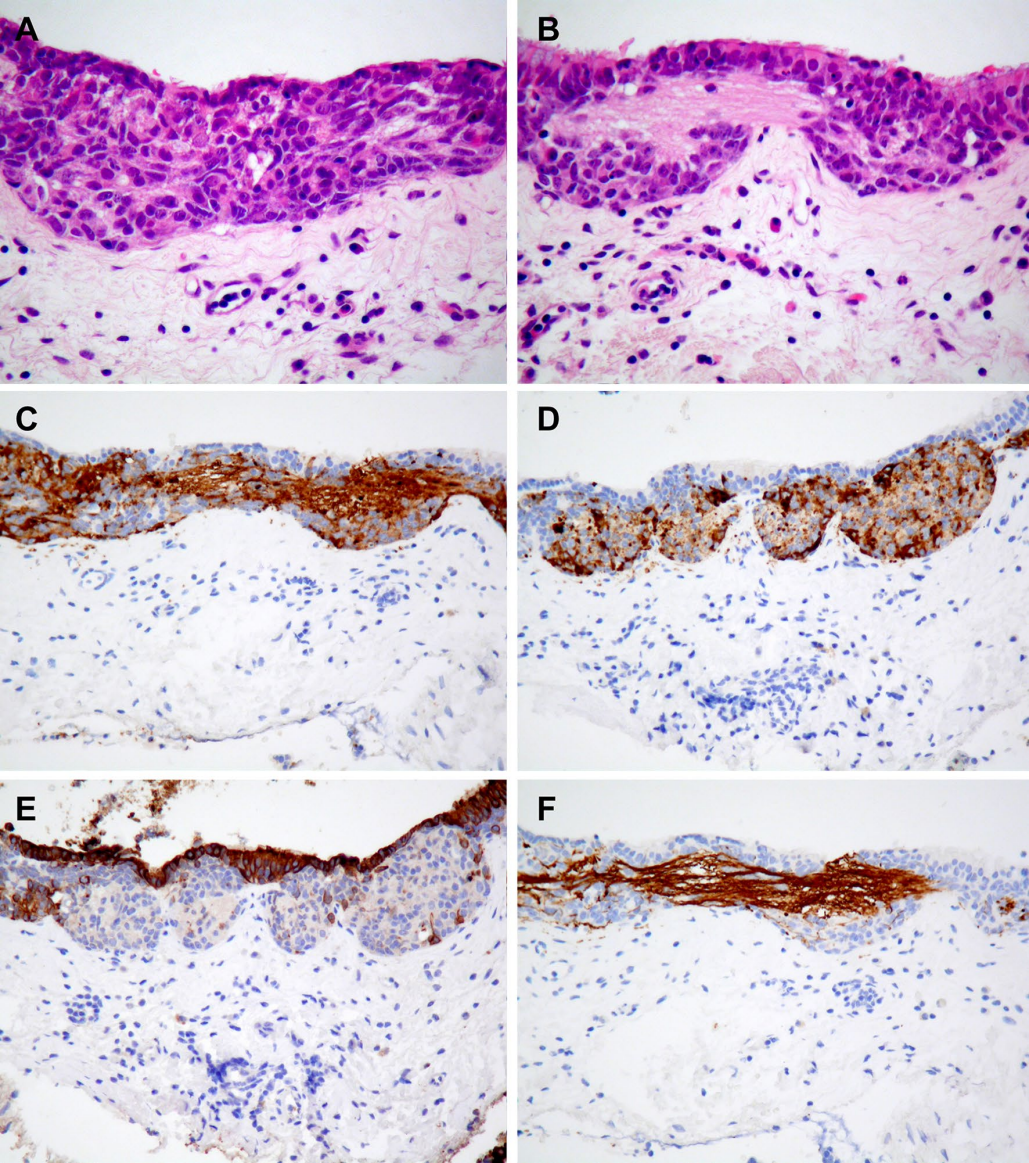
Biopsy specimen showing microscopic and IHC features of in situ neuroendocrine cell hyperplasia. **a** Sinonasal mucosa of left olfactory groove with hyperplastic neuroendocrine cell nests with **b** fibrillary neuropil-like substance (H-E, × 200). Sinonasal mucosa of left olfactory groove stained with **c** synaptophysin, **d** chromogranin, **e** cytokeratin and **f** neurofilament showing neuropil-like substance (IHC, x200)

Histopathological analysis of the biopsy specimen showed respiratory epithelium with in situ neuroendocrine cell hyperplasia, which was in the form of cell nests composed of small cells containing uniform nuclei and small nucleoli, with interspersed fibrillary neuropil-like material (Fig. 2a, b). The cells stained with chromogranin and synaptophysin, and the neuropil-like material was positive for neurofilament (Fig. 2c–f). No neuroblastoma or neuroendocrine carcinoma were noted in the biopsy. The patient was offered radical resection by an image-guided bicoronal craniotomy and transnasal endoscopic approach. The lateral borders of tumour were dissected to the skull base and anterior and posterior bony borders were drilled down, before repair was made using a right nasoseptal flap. Tumour resection was then completed intracranially.

**Fig. 3:**
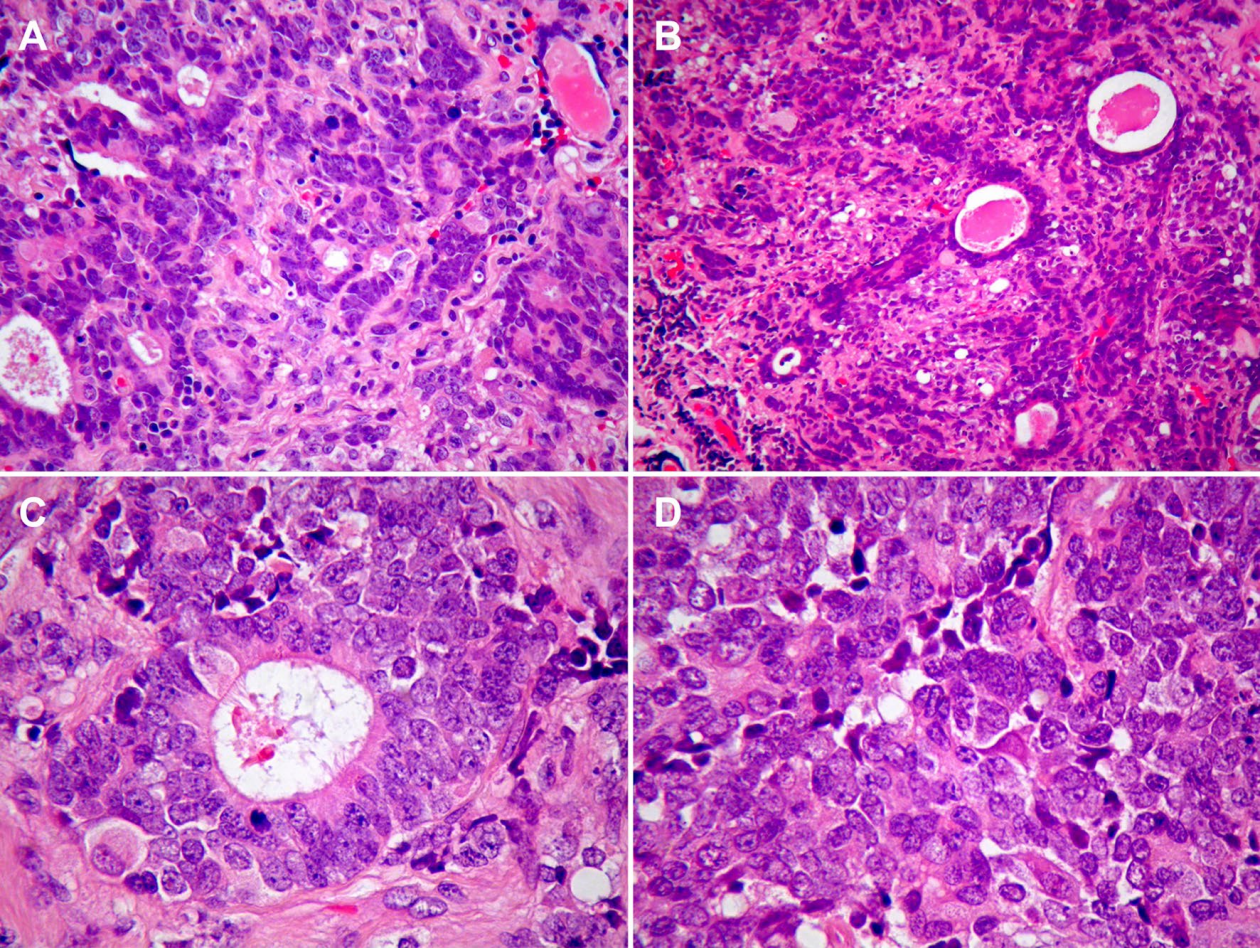
**a**, **b** Tumour resection specimen showing microscopic features of a mixed pattern tumour (H-E, **a**, **b** x200). **(c)** Adenocarcinoma component and **(d)** ONB component (H-E, x400)

The specimens surgically resected from the left sinonasal cavity and from inside the anterior cranial fossa revealed a distinctly biphasic tumour, comprising an epithelial component of atypical cuboidal and ciliated columnar cells, and a neuroendocrine component of primitive-appearing small cells with scant cytoplasm and speckled nuclear chromatin (Fig. 3a–d).

The epithelial component was prominent within the sinonasal cavity, and formed glands, cords, cribriform structures and ill-defined sheets. The neuroendocrine component comprising small cells was interspersed with the glandular element. In the intracranial specimen, ill-defined and anastomosing sheets of both components were present. Brain tissue was identified and it is likely that cells with moderately abundant eosinophilic cytoplasm represented native neuronal cells rather than ganglionic differentiation within the tumour. No focal necrosis was seen, however, apoptotic nuclear fragments were present amongst tumour cells. There were, on average, two mitoses per ten high power fields.

**Fig. 4:**
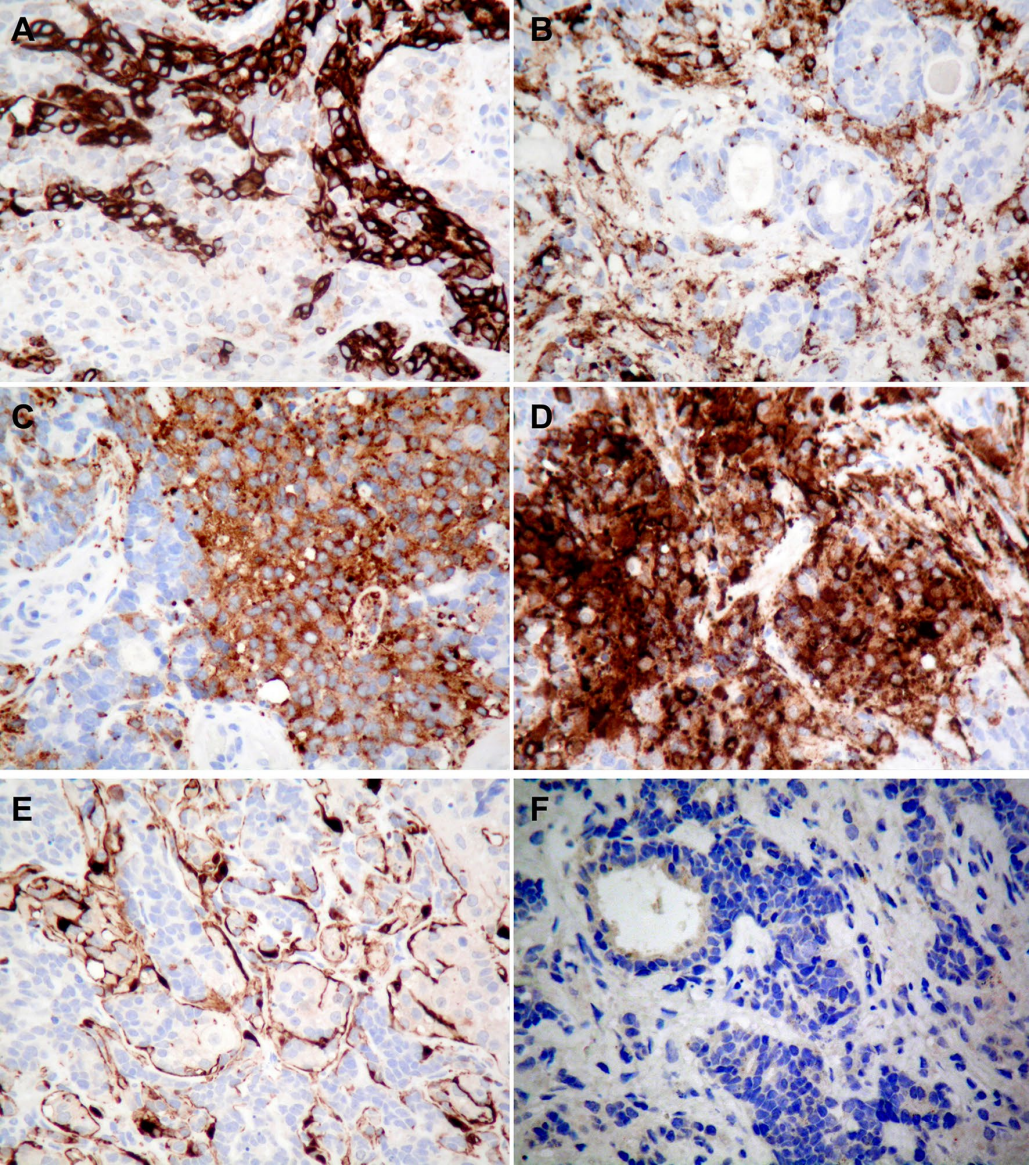
Tumour resection specimen showing IHC features of mixed ONB and adenocarcinoma. **a** Pancytokeratin staining of adenocarcinoma component. **b** Chromogranin, **c** synaptophysin, **d** calretinin, and **e** S100 IHC of neuroendocrine cells. **f** Negative p63 IHC staining in both adenocarcinoma and ONB components (IHC, **a**–**f**, x200)

Immunohistochemical analysis of the epithelial component showed strong cytokeratin CAM 5.2, AE1/AE3 and CK positivity (Fig. 4a). The small neuroendocrine cells showed patchy and variable intensity perinuclear staining at least focally. Epithelial membrane antigen stained the epithelial component and there was a diffuse, dot-like staining of the neuroendocrine cells. CD56, chromogranin, synaptophysin and calretinin stained the neuroendocrine cells but not the epithelial component (Fig. 4b–d). On S100 staining, there was a focal peripheral staining of some tumour groups in the intracranial component in a manner similar to that observed with sustentacular cells in ONB (Fig. 4e). There was no staining with p63 (Fig. 4f). The proliferation fraction was variable but low overall, and estimated to be less than 10%.

In summary, this was a biphasic tumour with epithelial and neuroendocrine components. Sinonasal teratocarcinosarcoma is a rare tumour that can arise in this site and shows a combination of epithelial, mesenchymal and neuroepithelial elements. This entity was not considered in the differential diagnosis due to the lack of heterologous epithelial elements including squamous cells and of a mesenchymal component with rhabdomyoblastic, cartilaginous, osteoblastic, smooth muscle or adipocytic differentiation [17]. Based on the morphology and immunophenotype, the following diagnoses were considered: combined neuroendocrine carcinoma and adenocarcinoma, ONB with divergent glandular differentiation, and mixed ONB and adenocarcinoma. The diagnosis of ONB was favoured over a neuroendocrine carcinoma due to the presence of neuropil-like material, S100 positive sustentacular cells focally within the tumour, positivity of the neuroendocrine component for calretinin and negativity for p63, location of the lesion arising out of the left olfactory groove, and the presence of neuroendocrine hyperplasia. The last feature has been described in ONB [18]. The distinction between co-incident adenocarcinoma and divergent glandular differentiation, both of which have been described in cases of ONB, was made on the basis of significant cellular atypia, ciliated cells, and irregular CK staining in the glandular component. Following these considerations, a final diagnosis of mixed ONB and adenocarcinoma was made.

The patient went on to receive adjuvant chemotherapy and radiotherapy. Post-treatment MRI head and whole-body positron emission tomography (PET)-CT showed no evidence of metastasis. However, 9 months after treatment, a soft mobile mass measuring 2 cm in the left neck level IIb/III was identified. Ultrasound of the neck revealed bilateral level IIb-IV nodes. Fine needle aspiration of the palpable left node was performed, which was highly cellular and contained malignant cells arranged both in groups and as a dispersed population. Some cells showed high nuclear-to-cytoplasmic ratio, whilst others contained moderate amounts of pale blue cytoplasm. In places the nuclei had speckled chromatin and showed evidence of moulding. Mucoid material with scattered macrophages and lymphoid cells were present in the background. This cytomorphology was considered consistent with metastatic spread of the primary tumour.

The patient underwent bilateral modified radical neck dissection and node clearance with a temporary tracheostomy for 1 year. Histological examination confirmed metastatic adenocarcinoma with occasional small areas of solid growth suggestive of neuroendocrine differentiation in 20 of 115 nodes. The patient received adjuvant radiotherapy for 6 weeks. Six month post-treatment whole-body PET-CT has shown no evidence of recurrent disease.

## Discussion

The relatively small number of cases of mixed ONB that have been described to date means that diagnosing and distinguishing mixed ONB from ONB with divergent differentiation and combined neuroendocrine carcinoma remains a significant challenge for the pathologist [19].

The demographic profile of this patient was consistent with the epidemiology of typical ONB. The clinical presentation of a first tonic–clonic seizure was unusual and contributed to by intracranial extension and possibly also moderate hyponatraemia. The nine-month history of episodic headaches and anosmia is consistent with the average time between symptom onset to diagnosis which is reported in the literature for typical ONB, and underlines the insidious nature of the clinical presentation of this tumour.

In this case, the diagnosis of ONB was made on the basis the following features. There was tumour positivity with calretinin and absent staining with p63 [14]. Calretinin is positive in 95% of cases of ONB [20] and p63 is negative in 76% of ONB cases [21]. While a minority of cases of neuroendocrine carcinoma and small cell carcinoma show some positivity for calretinin [20], in this case, low staining for MIB, a low mitotic index, and relatively low levels of necrosis and apoptosis makes these diagnoses unlikely [22]. In addition, the diagnosis of ONB is supported by what we consider represents in situ neuroendocrine hyperplasia within surface epithelium [23] including neuropil-like material, and S100 positive sustentacular cells focally within the tumour. Clinically, the lesion appeared to be arising in the left olfactory groove.

The glandular component in this case represented an adenocarcinoma rather than divergent glandular differentiation, as there was focal but significant atypia [24], and much of the glandular tissue comprised ciliated cells, whereas true Flexner-Wintersteiner rosettes, found in about 5% of ONB, have a lining of non-ciliated cells. In addition, irregular islands of CK-staining epithelial cells were in keeping with adenocarcinoma.

The changes seen in the lining of the respiratory epithelium (Fig. 4) were unusual and raised two possibilities: intraepithelial spread of tumour or neuroendocrine cell hyperplasia representing in situ change. Although it is difficult to differentiate between these two morphologically, there was no underlying invasive tumour in the biopsy where these changes were observed; intraepithelial spread is mostly seen in conjunction with subepithelial tumour. Hence, we believe that this represents sampling of a preserved early phase of the disease that has been theorised; however, by the time the tumour reaches clinical attention, in situ disease alone can no longer be appreciated [22]. To the best of our knowledge, this type of epithelial neuroendocrine cell hyperplasia associated with neuropil-like fibrillary processes has not been documented alongside neuroblastoma in the literature before, and lends support to the theory that ONB arises from neural stem cells in the olfactory neuroepithelium.

Whether mixed ONB cases behave more or less aggressively than typical ONB is not clear, making the selection of optimal treatment strategies and prognostication a clinical dilemma [25]. An analysis of six cases of mixed ONB has shown that tumours with epithelial divergence are associated with a higher grade ONB component than those with ganglioneuronal differentiation [13]. In our patient, lymph node metastasis was detected within 9 months of primary surgery, despite adjuvant chemotherapy and radiotherapy. This suggests that when ONB is mixed with an adenocarcinoma, it shows an aggressive clinical behaviour compared to that of typical ONB. Therefore, these uncommon subtypes require more aggressive treatment and follow-up compared to typical ONB. The patient is alive 2 years after initial diagnosis, but the prognosis remains uncertain. It is hoped that documentation of such rare cases will help a future better understanding of its clinical behaviour and hence treatment.

## Conclusion

ONB is an uncommon malignant neoplasm which makes up a small but significant proportion of head and neck cancers. We present a rare case of mixed ONB with adenocarcinoma with intraepithelial neuroendocrine cell hyperplasia in the olfactory groove. This finding provides a unique insight into the cellular origin of this rare tumour, and appears to confirm the theory that ONB arises from neural stem cells in the olfactory neuroepithelium. Despite surgical resection and adjuvant chemoradiotherapy, this tumour metastasised to cervical nodes, emphasising its aggressive clinical nature.
